# Identification of the c‐Jun/H19/miR‐19/JNK1 cascade during hepatic stellate cell activation

**DOI:** 10.1002/ctm2.1106

**Published:** 2023-03-02

**Authors:** Ying Sun, Chunyu Liu, Xu Guo, Jiayu Zhao, Anqi Xiao, Kai Yin, Ming Liu, Xinlei Sun, Xi Chen, Minghui Liu

**Affiliations:** ^1^ State Key Laboratory of Pharmaceutical Biotechnology Collaborative Innovation Center of Chemistry for Life Sciences Jiangsu Engineering Research Center for MicroRNA Biology and Biotechnology NJU Advanced Institute for Life Sciences (NAILS) School of Life Sciences Nanjing University Nanjing Jiangsu China; ^2^ School of Life Science and Technology China Pharmaceutical University Nanjing Jiangsu China

To the Editor:

Activation of hepatic stellate cells (HSCs) is considered as the central event during liver fibrosis.^1^, ^2^ H19 is an imprinted transcript and is upregulated during cholestatic liver fibrosis.^3^, ^4^, ^5^, ^6^ However, few studies have explored the regulatory network of H19 during HSC activation. Here, we report that c‐Jun, H19, miR‐19a/b‐3p and JNK1 form a feedback loop to promote HSC activation and hepatic fibrosis. Through fluorescence in situ hybridisation (FISH) and immunofluorescence (IF) assays, we observed the overexpression of H19 in both BDL‐induced and CCL4‐induced fibrotic livers (Figure S1) and the colocalisation of H19 with α‐smooth muscle actin (α‐SMA), a protein marker of activated HSCs (Figure 1B). In addition, H19 was upregulated in the transforming growth factor β1 (TGFβ1)‐activated human HSC cell line LX‐2 cells (Figure 1C). Upregulation of H19 further exacerbated LX‐2 activation (Figure 1D–F and Figure S2A–C), whereas downregulation of H19 reduced the protein levels of α‐SMA and collagen 1 in activated LX‐2 cells (Figure S2D–F).

**FIGURE 1:**
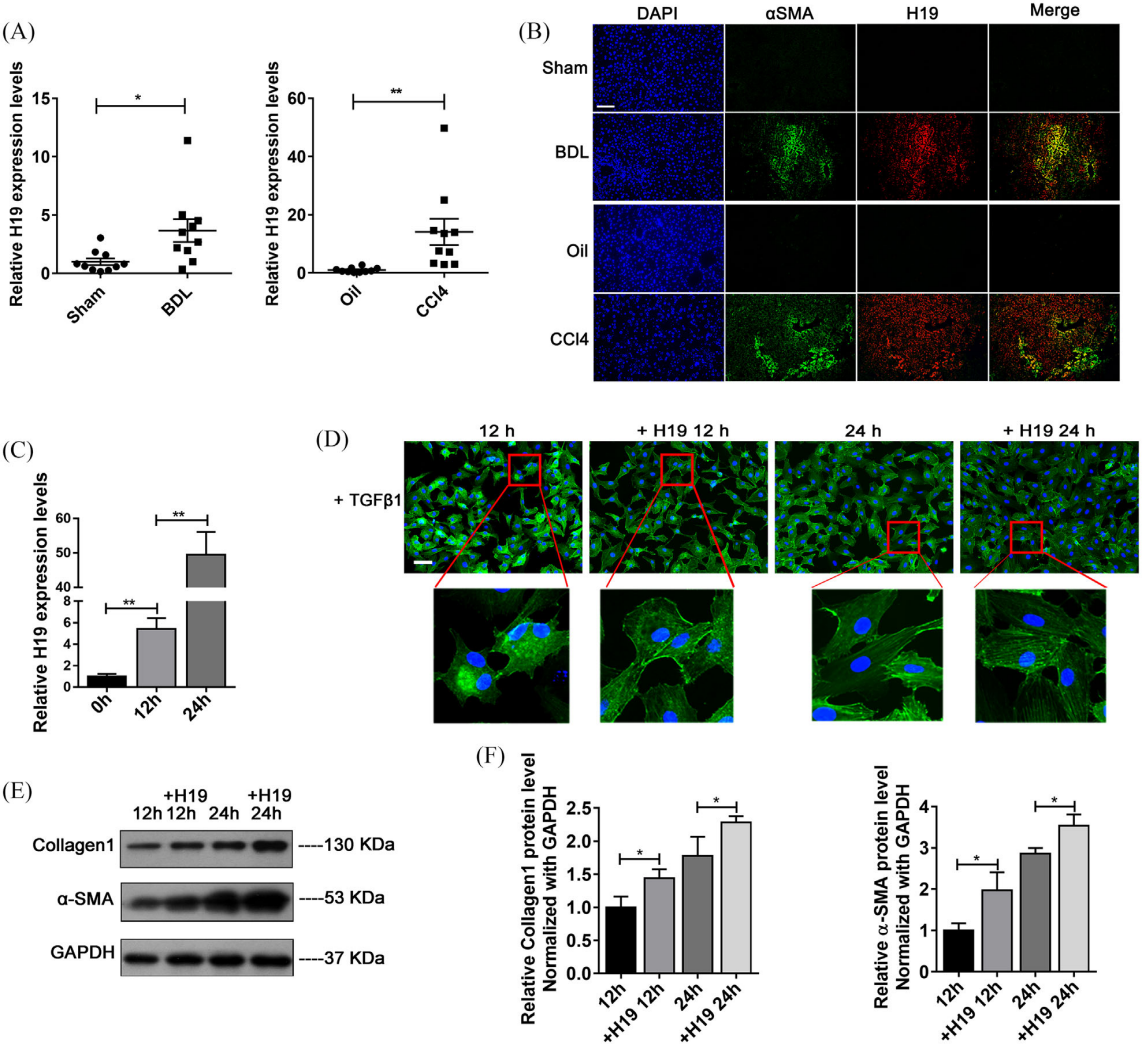
H19 is upregulated in fibrotic livers and activated LX‐2 cells and colocalises with activated HSCs. (A) Quantitative RT–PCR analysis of H19 lncRNA levels in liver fibrosis mouse models induced by BDL and CCL4. (B) FISH and IF assays: colocation of α‐SMA (as a marker of activated HSCs) and H19 in mouse fibrotic livers (magnification: ×200; scale bar: 50 μm). (C) Quantitative RT–PCR analysis of upregulated H19 levels in LX‐2 cells activated by TGFβ1 (10 ng/ml) for 0, 12 and 24 h. (D) Cytoskeleton staining by phalloidin suggested that upregulation of H19 promoted the fibrillation of LX‐2 cells. (magnification: ×200; scale bar: 50 μm). (E and F) Western blot analysis: H19 overexpression increased the protein levels of α‐SMA and collagen 1 in activated LX‐2 cells, suggesting an increased activation and fibrogenic activity of LX‐2 cells. Values are presented as the means ± SEMs. Significance was determined using two‐tailed Student's *t*‐test between two groups. *p < 0.05; **p < 0.01; ***p < 0.001

To explore how H19 is upregulated in activated HSCs, we searched for upstream transcription factors (TFs) that potentially target the H19 gene (Figure 2A). c‐Jun was found to be positively correlated with H19 (Figure S3) and has two potential binding sites at the promoter region of the H19 gene (Figure 2B). Luciferase reporter assays and ChIP assays verified the direct binding between the c‐Jun protein and the H19 promoter region (Figure 2C,D). Increased c‐Jun protein levels (Figure 2E–G) were also determined to be positively correlated with H19 levels in both BDL‐induced and CCL4‐induced fibrotic livers (Figure 2H). Overexpression of c‐Jun increased H19 levels in LX‐2 cells and vice versa (Figure 2I). These results suggested that c‐Jun could trigger H19 transcription by binding to the promoter region during HSC activation.

**FIGURE 2:**
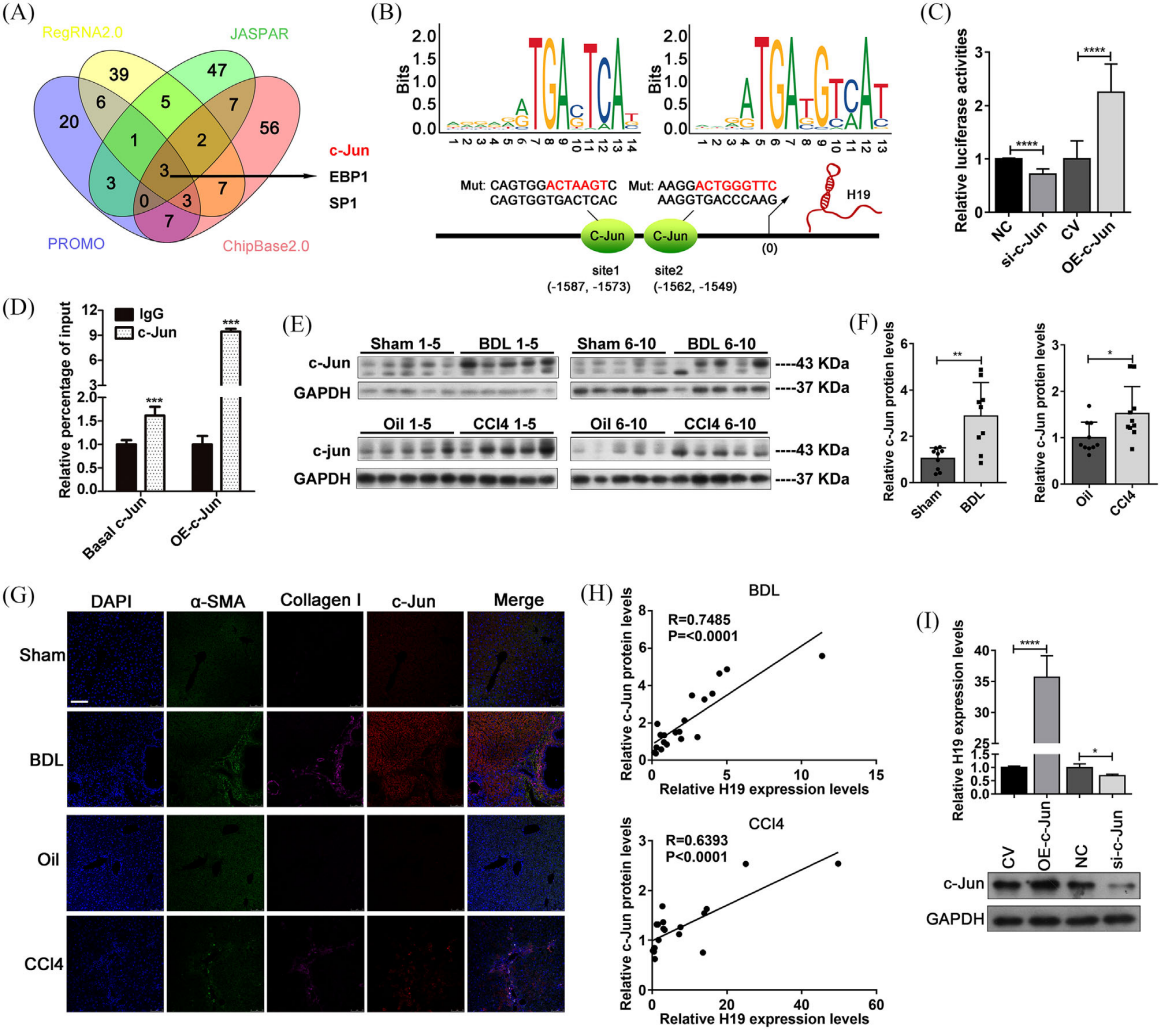
c‐Jun converges on the H19 promoter to facilitate its expression. (A) Venn diagram analysis of TFs that potentially targeted the H19 promoter region. (B) Potential binding sites of c‐Jun protein in the promoter region of the H19 gene. (C) Luciferase reporter gene assay demonstrated the direct binding between the c‐Jun protein and the promoter region of the H19 gene. (D) Quantitative RT–PCR analysis of H19 gene after ChIP assay, which confirmed the direct binding between the c‐Jun protein and the H19 gene. (E and F) Western blot analysis of the c‐Jun protein levels in liver fibrosis mouse models induced by BDL and CCL4. (G) IF staining for α‐SMA (green), collagen 1 (pink), c‐Jun (red) and DAPI (blue) showed an accumulation of c‐Jun during liver fibrosis (magnification: ×200; scale bar: 50 μm)). (H) The positive correlation between c‐Jun protein levels and H19 levels in fibrotic livers induced by BDL and CCL4. (I) Overexpression of c‐Jun in LX‐2 cells increased H19 expression, and downregulation of c‐Jun decreased H19 levels. Values are presented as the means ± SEMs. Significance was determined using two‐tailed Student's *t*‐test between two groups. *p < 0.05; **p < 0.01; ***p < 0.001; ****p < 0.0001

Given that H19 was mainly located in the cytoplasm of LX‐2 cells (Figure 3A), we speculated that H19 might function as a competitive endogenous RNA (ceRNA). We screened eight miRNAs potentially sponged by H19 using bioinformatic analysis (Figure 3B). Among them, significant enrichment of miR‐19a/b‐3p was observed in the RNA samples pulled down by biotin‐labelled H19 probe (Figure 3C and Figure S4A), indicating direct binding between miR‐19a/b‐3p and H19, which was also confirmed by a luciferase reporter assay (Figure 3D,E). However, H19 expression did not inhibit miR‐19a/b‐3p levels in LX‐2 cells (Figure S5), suggesting that H19 might hijack miR‐19a/b‐3p to affect its function but not its expression.

**FIGURE 3:**
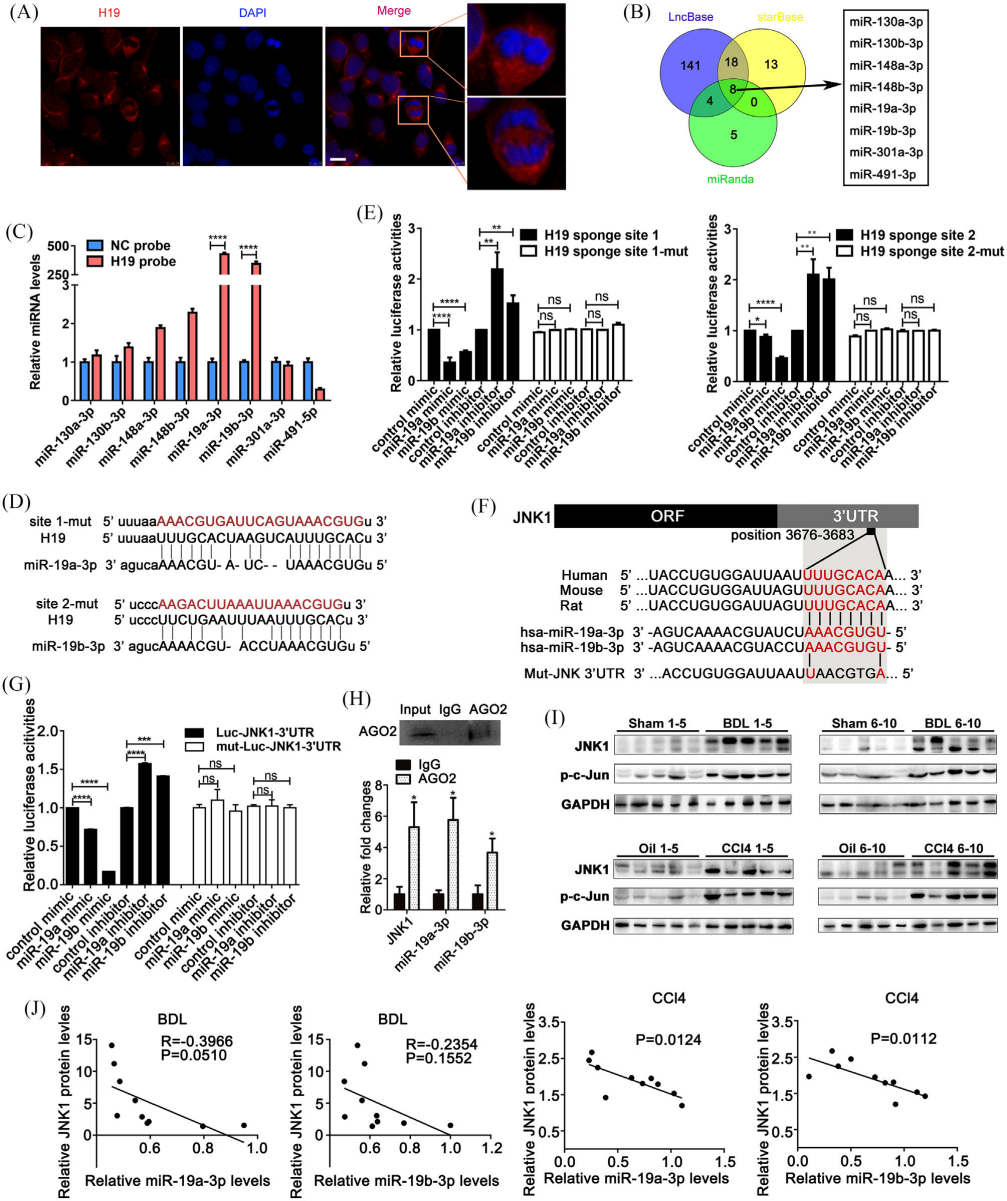
H19 acts as a sponge of miR‐19a/b‐3p in activated HSCs to remove the inhibition of miR‐19a/b‐3p on the fibrogenic factor JNK1. (A) Subcellular location of H19 in LX‐2 cells (magnification: ×400; scale bar: 10 μm). (B) Bioinformatic analysis: miRNAs potentially sponged by H19. (C) Enrichment of miR‐19a/b‐3p in the pulldown samples by a biotin‐labelled H19 probe. (D) Complementary binding sites were predicted between miR‐19a/b‐3p and H19. (E) Luciferase reporter gene assays evaluated the direct binding between H19 and miR‐19a/b‐3p. (F) The predicted binding sites between miR‐19a/b‐3p and the JNK1 3′UTR. (G and H) Luciferase reporter gene assays and RIP assays demonstrated the direct binding between miR‐19a/b‐3p and the JNK1 3′UTR. (I) Western blot analysis of the JNK1 protein levels and phosphorylated c‐Jun (p‐c‐Jun) protein levels in fibrotic livers induced by BDL and CCL4. (J) The negative correlation between JNK1 protein levels and miR‐19a/b‐3p levels. Values are presented as the means ± SEMs. Significance was determined using two‐tailed Student's *t*‐test between two groups. *p < 0.05; **p < 0.01; ***p < 0.001; ****p < 0.0001; ns, not significant

Although miR‐19b has been reported to display an inhibitory effect in HSC‐mediated fibrogenesis,^7^ the underlying mechanism is yet to be fully explained. We found that c‐Jun N‐terminal kinases 1 (JNK1) is potential target gene of miR‐19a/b‐3p. Up to eight bases of complementary pairing were found between the 3′ untranslated region (3′UTR) of JNK1 and the seed sequences of miR‐19a/b‐3p (Figure 3F). A luciferase reporter assay confirmed that miR‐19a/b‐3p directly targeted the JNK1 3′UTR (Figure 3G). RNA immunoprecipitation (RIP) assay detected that the AGO2 protein binds both miR‐19a/b‐3p and JNK1 mRNA (Figure 3H). In LX‐2 cells, JNK1 protein expression was suppressed by miR‐19a/b‐3p overexpression and enhanced by miR‐19a/b‐3p knockdown (Figure S4B,C). In both BDL‐induced and CCL4‐induced fibrotic livers, the levels of JNK1 protein and its phosphorylation substrate,^8^, ^9^ and phosphorylated c‐Jun (p‐c‐Jun) were significantly enhanced (Figure 3I and Figure S4D). JNK1 protein levels were inversely correlated with miR‐19a/b‐3p (Figure 3J). Previous studies have reported that JNK signaling is crucial for cell death, survival, differentiation, proliferation and tumorigenesis in the liver.^10^ The activation of JNK1 in HSCs is an essential fibrogenic event during hepatic fibrosis.^8^ Our data suggested that miR‐19a/b‐3p played an inhibitory role in liver fibrosis at least partly by negatively regulating JNK1 in HSCs.

Next, we confirmed the downregulation of miR‐19a/b‐3p in clinical liver diseases by analysing sequencing data from the GEO database, including hepatitis C virus (HCV)‐infected livers, hepatitis B virus (HBV)‐infected livers, alcoholic steatohepatitis and nonalcoholic steatohepatitis (NASH) (Figure 4A). Reduced miR‐19a/b‐3p levels were also detected in the mouse fibrotic livers (Figure 4B). We further determined the inhibitory role of miR‐19a/b‐3p during hepatic fibrosis by lentivirus‐mediated miR‐19a/b‐3p overexpression (Figure S6A). Compared to the control lentivirus groups, the miR‐19a/b‐3p‐overexpressing lentivirus significantly restored the fibrotic phenotypes and the hepatic function indices in both BDL‐induced and CCL4‐induced livers (Figure 4C–E and Figure S6B,C).

**FIGURE 4:**
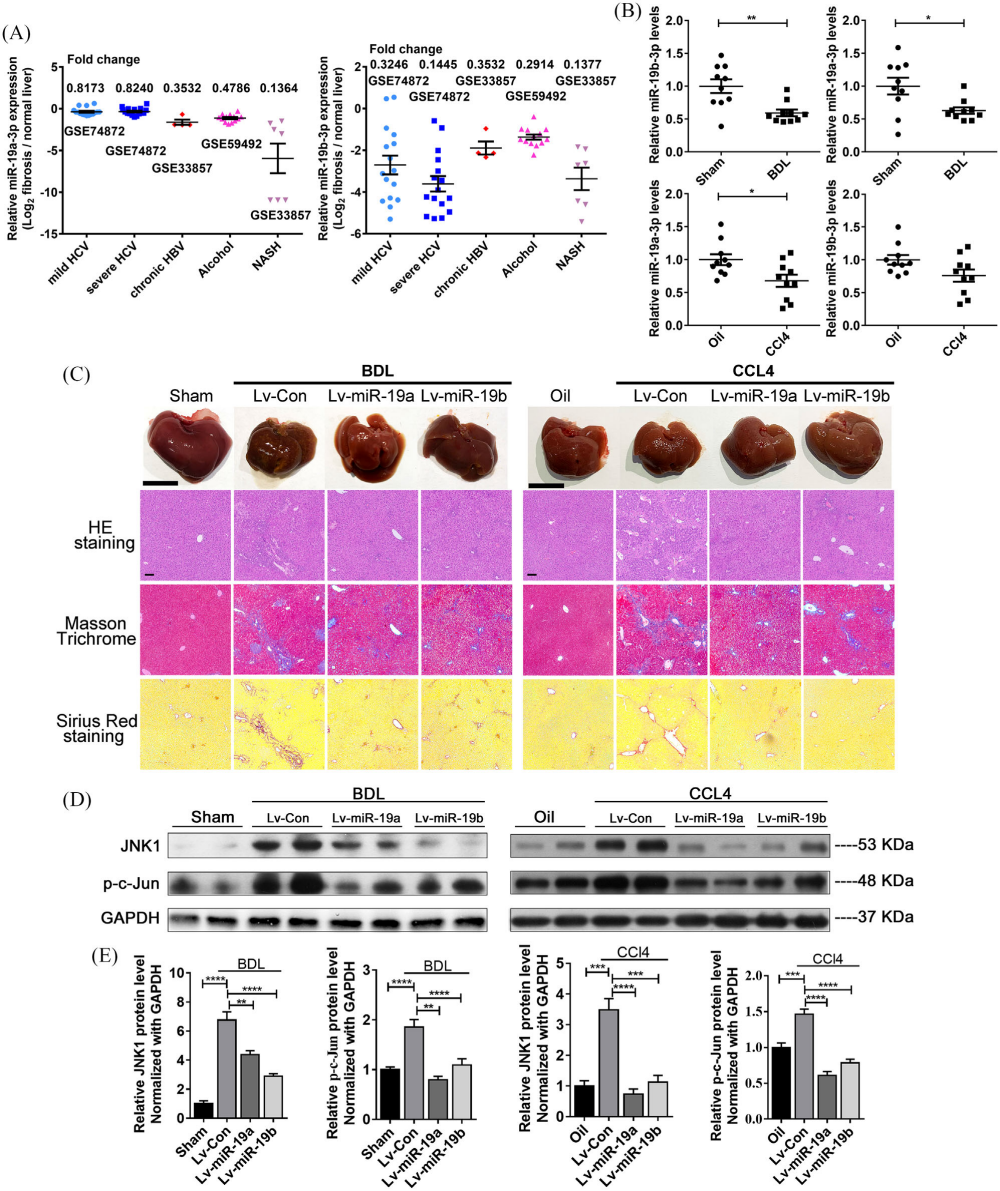
miR‐19a/b‐3p reversed liver fibrosis in both the BDL and CCL4‐induced mouse models. (A) GEO data analysis: miR‐19a/b‐3p levels are downregulated in hepatitis C virus (HCV)‐infected liver, hepatitis B virus (HBV)‐infected liver, alcoholic steatohepatitis and nonalcoholic steatohepatitis (NASH). (B) miR‐19a/b‐3p levels in fibrotic livers induced by BDL surgery and CCL4. (C) Histomorphology of fibrotic livers with or without miR‐19a/b‐3p overexpression. Scale bar for white light pictures: 1 cm; scale bar for pathological sections: 100 μm (magnification: ×100). (D and E) Western blot analysis of JNK1 and phosphorylated c‐Jun (p‐c‐Jun) protein levels in fibrotic livers induced by BDL and CCL4 after treatment with or without miR‐19a/b‐3p‐overexpressing lentivirus. Values are presented as the means ± SEMs. Significance was determined using two‐tailed Student's *t*‐test between two groups. *p < 0.05; **p < 0.01; ***p < 0.001; ****p < 0.0001; ns, not significant

Herein, we identified a regulatory loop consisting of c‐Jun/H19/miR‐19a/b‐3p/JNK1/c‐Jun during HSC activation. In detail, when the liver is injured, H19 levels in HSCs were increased along with rapid propagation of HSCs. Previous research showed that both quiescent HSCs and activated HSCs could absorb H19 derived from cholangiocytes in cholestatic liver disease (Figure S7).^6^ Our study demonstrated that overexpression of H19 in HSCs, at least partly, also depends on c‐Jun‐triggered transcription during liver fibrosis. In turn, increased H19 in HSCs can relieve the inhibition of miR‐19a/b‐3p on JNK1 and thereby enable JNK1 to activate c‐Jun, which will further promote the expression of H19. This cascade points out a new mechanism of HSC activation and transition from the initial activation state to the permanent activation state, and reinforces the value of epigenetic regulation during hepatic fibrosis. The in vivo results of lentivirus‐mediated miR‐19a/b‐3p overexpression restoring the fibrotic phenotypes of both BDL and CCL4 mouse models also provide proof for miRNA‐targeted therapies as novel therapeutic strategies for the prevention and treatment of liver fibrosis.

## CONFLICT OF INTEREST

The authors declare that they have no conflicts of interest.

## Supporting information

Supporting Material

Supporting Material

Supporting Material

Supporting Material

Supporting Material

Supporting Material

Supporting Material

Supporting Material
