# Supplementary material for: Identification of the c‐Jun/H19/miR‐19/JNK1 cascade during hepatic stellate cell activation
Source: Clin Transl Med. 2023 Mar 2;13(3):e1106. doi: 10.1002/ctm2.1106 (PMC9982076; doi:10.1002/ctm2.1106)
Supplement: Supplementary file 1 — Supporting Material [file CTM2-13-e1106-s006.docx]

**Supplementary Materials for**

**Identification of the c-Jun/H19/miR-19/JNK1 cascade during hepatic stellate cell activation**

*Ying Sun^1^,* *Chunyu Liu^1^, Xu Guo^1^, Jiayu Zhao^1^, Anqi Xiao^1^, Kai Yin^1^, Ming Liu^1*^, Xinlei Sun^1*^, Xi Chen**^1*^ and Minghui Liu^1,2*^*

*** Corresponding authors:**

Minghui Liu, minghuiliu@cpu.edu.cn;

Xi Chen, [xichen@nju.edu.cn;](mailto:xichen@nju.edu.cn;)

Ming Liu, [liuming_nju@163.com](mailto:liuming_nju@163.com);

Xinlei Sun, s627016776@sina.com

**This file includes:**

Supplementary Figures and Figure Legends (Sup-Figure 1 ~ Sup-Figure 7)

Supplementary Materials and Methods

Supplementary Table 1

Supplementary Discussion

Supplementary Reference

**SUPPLEMENTARY FIGURES AND** **FIGURE LEGENDS**

**Sup-Figure 1. Successful induction of mouse fibrotic liver models by BDL and CCL4.**

**(A)** Histomorphology (HE staining, Masson’s trichrome staining and Sirius Red staining) of BDL and CCL4-induced mouse fibrotic livers. Scale bar for white light pictures, 1 cm; scale bar for pathological sections, 100 μm (magnification, ×100) **(B)** Pathological statistics of fibrotic livers (ND: not detect). **(C)** The levels of the hepatic Hyp, serum AST and serum ALT in fibrotic livers. **(D and E)** Increased α-SMA and collagen 1 protein levels confirmed the successful construction of mouse fibrotic livers. Values are presented as the means ± SEMs. Significance was determined using two-tailed Student’s t test between two groups. *P < 0.05; **P < 0.01; ***P < 0.001; ****P < 0.0001; ns, not significant.

**Sup-Figure 2. Overexpression of H19 in TGFβ1-treated LX-2 cells further exacerbates HSC activation.**

**(A and B)** Quantitative RT–PCR analysis: H19 overexpression increased the mRNA levels of α-SMA and collagen 1 in activated LX-2 cells, suggesting an increased fibrogenic activity of LX-2 cells. **(C)** Quantitative RT–PCR analysis: The H19 overexpressing plasmid successfully upregulated the level of H19 in LX-2 cells. **(D and E)** Western blot analysis: Knockdown of H19 level reduced the protein levels of α-SMA and Collagen 1 in activated LX-2 cells. **(F)** Quantitative RT–PCR analysis: The specific H19 siRNA (si-H19) successfully downregulated the level of H19 in LX-2 cells compared to control siRNA (si-NC). Values are presented as the means ± SEMs. Significance was determined using two-tailed Student’s t test between two groups. *P < 0.05; **P < 0.01; ***P < 0.001; ****P < 0.0001; ns, not significant.

**Sup-Figure 3. ChIPBase database predicted the correlation between the potential TFs (c-Jun, EBP1 and SP1) levels and H19 levels in the liver.**

We searched for upstream transcription factors that potentially target the H19 gene using miRwalk2.0, JASPAR, PROMO and ChIPBase. Three genes i.e., c-Jun, EBP1 and SP1 were screened out and c-Jun was finally found to be significantly correlated with H19 through Pearson’s correlation analysis using the ChIPBase database.

**Sup-Figure 4. H19 acts as a sponge of miR‐19a/b-3p in activated HSCs to remove** **the inhibition of miR-19a/b-3p on the fibrogenic factor JNK1.**

**(A)** RNA pulldown assay: H19 was successfully pulled down with a biotin-labeled H19 probe in LX-2 cells. **(B)** Quantitative RT–PCR analysis of miR-19a-3p and miR-19b-3p levels in LX-2 cells transfected with miR-19a/b-3p mimics or inhibitors. **(C)** Western blot analysis showed an inhibitory effect of miR-19a/b-3p on JNK1 expression. **(D)** Western blot analysis of JNK1 protein levels and phosphorylated c-Jun (p-c-Jun) protein levels in fibrotic livers induced by BDL and CCL4 (corresponding to **Figure 3I**). Values are presented as the means ± SEMs. Significance was determined using two-tailed Student’s t test between two groups. *P < 0.05; **P < 0.01; ***P < 0.001; ****P < 0.0001; ns, not significant.

**Sup-Fig****ure 5. H19 levels do not affect the expression levels of miR-19a/b-3p.**

LX-2 cells were transfected with the H19 overexpression vector or H19 siRNA, Twenty-four hours later, total RNA was isolated and the H19 lncRNA and miR-19a/b-3p levels were tested by quantitative RT–PCR. H19 overexpression vector dose-dependently increased H19 expression **(A)** and H19 siRNA transfection dose-dependently attenuated the H19 levels **(B)**, but the levels of miR-19a/b-3p were not affected by the changes in H19 expression levels in LX-2 cells. Values are presented as the means ± SEMs. Significance was determined using two-tailed Student’s t test between two groups. *P < 0.05; **P < 0.01; ***P < 0.001; ****P < 0.0001; ns, not significant.

**Sup-Figure 6. Overexpression of miR-19a/b-3p by lentivirus reversed liver fibrosis in both the BDL and CCL4-induced mouse models.**

**(A)** The successful overexpression of miR-19a/b-3p in the liver by tail vein injection of miR-19a/b-3p-expressing lentivirus. Compared to the control lentivirus (Lv-Con) groups, Lv-miR-19a and Lv-miR-19b successfully overexpressed miR-19a/b-3p in fibrotic livers induced by BDL or CCL4. **(B)** Pathological statistics of the BDL and CCL4-induced fibrotic livers treated with miR-19a/b-3p-overexpressing lentivirus or control lentivirus (corresponding to Figure 4C). **(C)** miR-19a/b-3p overexpression significantly reduced the levels of hepatic Hyp, serum AST and serum ALT in the BDL and CCL4-induced fibrotic livers. Values are presented as the means ± SEMs. ND, not detected. Significance was determined using two-tailed Student’s t test between two groups. *P < 0.05; **P < 0.01; ***P < 0.001; ****P < 0.0001.

**Sup-Figure 7** **Representative images of fluorescence in situ hybridization (FISH) targeting H19 and immunofluorescence (IF) of a cholangiocyte marker (CK-19) in the BDL-induced mouse model.** Colocalization of CK-19 (green) and H19 (red) is indicated by white arrows. CK-19 labeled cholangiocytes are indicated by the white square. Scale bar, 50 μm. PV, portal vein; yellow *, bile duct in the liver.

**SUPPLEMENTARY MATERIALS AND METHODS**

**Cells and reagents**

The human hepatic stellate cell line LX2 was purchased from the Shanghai Institute of Cell Biology, Chinese Academy of Sciences (Shanghai, China) and cultured in Dulbecco’s modified Eagle’s medium (DMEM, C11995500BT, Gibco), containing 10% FBS (A3160802, Gibco) and 1% Penicillin-streptomycin solution (30-002-CI, CORNING), in a 5% CO2 water-saturated and 37 °C atmosphere. siRNAs designed to specifically silence miR-19a, miR-19b, H19 and c-Jun were purchased from GenePharma (Shanghai, China), and a scrambled siRNA served as a control. The siRNA sequences are listed in the **supplementary Table 1**. Transient transfection of plasmids and siRNAs were performed using Lipofectamine 2000 (11668-019, Invitrogen) according to the manufacturer’s instructions. Total RNA and protein were isolated 24h and 48h after transfection, and were assessed by quantitative RT-PCR and western blotting, respectively. Adenovirus for overexpressing miR-19a and miR-19b were provided by HanBio (HanBio, Shanghai, China). Luciferase constructs were obtained from Genscript (Nanjing, China).

**Animal studies**

Wild type C57BL/6 male mice were purchased from the Model Animal Research Center of Nanjing University (Nanjing, China), and fed a standard rodent chow diet with free access to water, temperature-controlled (23±1°C), and pathogen-free facility under a 12h light/ dark cycle (light on 8 AM to 8 PM). For the chronic CCl4 study, mice were intraperitoneally injected with CCl4-olive oil solution (the dosage of CCl4 was 1 mL/kg) or pure olive oil twice a week (n=10 mice/group). After 4 weeks, the mice were sacrificed. For the BDL mouse models, mice were subjected to BDL or sham operation on day 1 and sacrificed after 2 weeks (n=10 mice/group). For in vivo viral transduction, mice were injected via the tail vein with purified Lv-miR-19a or Lv-miR-19b (HANBIO, Shanghai, China, HBLV-miR-19b) containing a thyroxine-binding globulin (TBG) promoter driving miR-19a or miR-19b gene expression at 1×10^7^ virus particles per mouse. For BDL mouse model, lentivirus treatment is on day 5 while CCL4 mouse model is at the beginning of the 3^rd^ week. After the molding is finished, all mice were sacrificed, and serum was collected to measure aspartate aminotransferase (AST) and alanine aminotransferase (ALT) activities. Liver tissues were harvested and either fixed and processed for histological analysis or frozen in liquid nitrogen and stored at −80°C for further analysis. The liver tissues were used for testing Hyp (hydroxyproline) concentrations by using commercially available assay kits (A030-2, JianCheng, Nanjing, China) according to the manufacturer’s instructions. Histological analysis including hematoxylin and eosin (HE) staining, Masson’s trichrome staining and Sirius red staining were performed (Servicebio, Wuhan, China), and six fields of each slide were randomly taken under microscope and quantified by ImageJ software. All mouse protocols followed the National Institutes of Health guidelines for the care and use of mice and were approved by the Institutional Animal Care and Use Committee of Nanjing University.

**RNA isolation and quantitative RT-PCR**

Total RNA was isolated from liver tissues and cultured cells using TRIzol Reagent (15596018, Ambion), and then reverse transcribed into cDNA using HiScript III RT SuperMix (R323-01, Vazyme) or miRNA Universal SYBR qPCR Master Mix (MR101, Vazyme). The H19 RNA or miRNA levels were determined by real-time RT-PCR (Q711 or MQ101, Vazyme) respectively, according to the manufacturer’s instructions on an Applied Biosystems 7300 Real-time PCR System (Applied Biosystems)

**Protein extraction and Western blot analysis**

Cells and liver tissues were harvested using RIPA Lysis and Extraction Buffer (89900, Thermo Scientific™) supplemented with PMSF Protease Inhibitor (36978, Thermo Scientific™). Determination of protein concentration and Western blotting were performed using selective antibodies and normalized to GAPDH. Protein bands were analyzed using ImageJ software.

**Histopathology, Masson’s trichrome staining and Sirius oil staining**

After being scarified, mouse liver tissues were collected, fixed with 4% formaldehyde and embedded in paraffin. Sections of 5 μm were stained with hematoxylin and eosin (H&E) staining, while liver fibrosis was determined by Masson’s trichrome staining and Sirius oil staining (Servicebio, Wuhan, China), and six fields of each slide were randomly taken under microscope and quantified by ImageJ software. The percentage of hyperchromatic areas (positive areas) in each field was calculated by ImageJ and then the average percentage of the six fields of each slide was taken as the final positive fraction to characterize the severity of liver fibrosis. The percentage of the positive fraction was proportional to the degree of liver fibrosis.

**Immunofluorescence** **(IF**)

After being sacrificed, mouse liver tissues were harvested, formalin-fixed and paraffin-embedded. Then liver sections were deparaffinized, rehydrated and blocked using 2% BSA PBS solution containing 0.1% Triton X-100. After incubation with primary antibodies targeting collagen I (14695-1-AP, proteintech), α-SMA (BM0002, BOSTER), c-Jun (9165, Cell Signaling Technology) and CK-19 (a cholangiocyte marker, 12434S, Cell Signaling Technology ) overnight at 4 °C, liver sections were washed and incubated with secondary antibodies with different fluorescence (Alexa Fluor^TM^ 488 donkey anti-mouse IgG(H+L), A21206, Alexa Fluor^TM^ 594 goat anti-rabbit IgG (H+L), A11012, Alexa Fluor^TM^ 647 goat anti-rabbit IgG (H+L), A32733, Invitrogen) in 5% BSA in a light-proof environment for 1 h at RT. Next, DAPI (C1006, Beyotime, Shanghai, China) was used for nuclear DNA counterstaining. Images were taken using a confocal microscope (Leica, Germany).

**Fluorescence in situ hybridization (FISH)**

In situ hybridization was carried out using biotin-labeled specific probes for lncRNA H19 (R11060.7, RiboBio, Shanghai, China) to detect the expression of H19 in HSCs and liver slices. LX-2 cells grown to the exponential phase on cell slides were subjected to cell fixation, probe detection and nuclear DNA counterstaining according to the kit instructions. For the purpose of colocalization with the fibrosis indicator α-SMA, liver tissue sections were first deparaffinized, rehydrated and hybridized in hybridization buffer with biotin-labeled lncRNA H19 probes at 37 °C overnight. Then, the liver sections were washed and incubated with primary α-SMA antibody (ab32575, Abcam) targeting α-SMA at 4 °C, and again, the liver sections were washed and incubated with fluorescent secondary antibody (GB22303, Servicebio, Wuhan, China). DAPI was used for nuclear DNA counterstaining. Finally, signals were measured by using a laser scanning confocal microscope (Leica, Germany) at 200x ~ 400x magnification.

**Relative luciferase activity detection.**

To test the promotive effect of the transcription factor c-Jun on lncRNA H19 expression, sequences containing the presumed c-Jun binding sites were designed from the promoter region of the H19 gene and inserted in front of the luciferase expressing sequence in reporter gene plasmid (PGL3-Basic-vector plasmid, Genescript, Nanjing, China). The c-Jun expression plasmid (OE-c-Jun) and the reporter gene plasmid were cotransfected into HEK293T cells. If c-Jun could activate the H19 promoter, the luciferase gene would be expressed, and the expression amount of luciferase was proportional to the action intensity of the transcription factor. To test binding specificity, sequences on the H19 genome that contained predicted c-Jun binding sites were mutated from TGATTCA to ACTAAGT and ACTGGGTTC to TGACCCAAG, and then the synthetic mutant fragment was also inserted into an equivalent reporter plasmid (Genescript, Nanjing, China). To determine the direct interaction between miR-19a/miR-19b and H19, the complementary binding sites between H19 and miR-19a-3p (site 1) as well as miR-19b-3p (site 2) were predicted by bioinformatics analysis. Then the sequences containing the predicted miR-19a and miR19b binding sites on the 3′-UTR of the H19 gene were inserted into the promoter region of the luciferase expressing gene in the PGL3-Basic-vector plasmid. After that, the plasmid was transferred into HEK293T cells together with miR-19a/miR-19b mimics or inhibitors, miR-19a/miR-19b mimics recognized and bound to the binding sites of H19 inserted to the promoter region of luciferase gene, and significantly reduced the luciferase protein expression. In contrast, miR-19a/miR-19b inhibitors prevented endogenous miR-19a/miR-19b binding to the binding sites, resulting in a moderate upregulation of the luciferase reporter gene. To test binding specificity, the sequences of H19 that interacted with miR-19a or miR-19b were mutated from A to T, G to C and vice versa, and the synthetic 3’-UTR mutant fragment was also inserted into an equivalent reporter plasmid as a control. The direct interaction between miR-19a/miR-19b and JNK1 was also determined by luciferase assays, in which sequences containing the presumed miR-19a and miR19b binding sites from the 3′-UTR of the JNK1 gene were inserted into the promoter area of luciferase protein gene in PGL3-Basic-vector plasmid (Genescript, Nanjing, China). To test binding specificity, sequences that interacted with the miR-19a or miR-19b were mutated from TTGCACA to AACGTGT, and the synthetic 3’-UTR mutant fragment was also inserted into an equivalent reporter plasmid. Luciferase reporter assays were performed as previously described [1]. HEK293T cells were cultured in 24-well plates, and each well was cotransfected with 0.2 μg of firefly luciferase reporter plasmid, 0.2 μg of β-galactosidase (β-gal) expression plasmid, and 50 pmol of mimics, inhibitors or scrambled negative control RNAs using Liposomal Transfection Reagent (40802ES03, Yeasen, Shanghai, China). The β-gal plasmid was used as a transfection efficiency control. The cells were mixed with the Luciferase Assay System (E1500, Promega, Madison, WI, USA) and assayed using the Turner BioSystems Instrument (9200-002,Turner BioSystems, Sunnyvale,CA).

**RNA pulldown**

Pierce™ Magnetic RNA-Protein Pull-Down Kit (20164, Thermo Scientific™) was used for RNA pull-down assays. LX-2 cells were harvested in lysis buffer (87788, Pierce) for 1 h on ice. Biotinylated H19 probe or control probe (Genescript, Nanjing, China) was incubated with streptavidin-coated magnetic beads (New England BioLabs, S1420S) for 1~2 h at room temperature to generate probe-coated magnetic beads. Then, whole-cell lysates were incubated overnight with probe-coated magnetic beads at 4 °C with constant rotation. The beads were collected using a magnetic frame and washed with buffer three times. RNA was further extracted with TRIzol reagent (15596018, Ambion, USA) and analyzed by reverse transcription-qPCR and real-time qPCR.

**Chromatin immunoprecipitation (ChIP)**

The ChIP assay was performed using a ChIP Assay Kit (P2078, Beyotime, Shanghai, China) according to the manufacturer’s instructions to research whether there is a direct interaction between c-Jun and the promoter region of the H19 gene by using standard ChIP procedures. The LX-2 cells were treated with 1% formaldehyde to cross-link the histones and genomic DNA, followed by ultrasonic lysis. Chromatin fractions with average sizes between 200-1000 bp were incubated with antibody against c-Jun (9165, Cell Signaling Technology), and the chromatin-antibody complexes were precipitated with Sepharose beads-protein G at 4 °C overnight. No antibody (input) and normal rabbit immunoglobulin G (IgG, Santa Cruz, USA) served as a negative control. Then chromatin DNA fragments were purified with a PCR purification kit (D0033, Beyotime, Nanjing, China), and subjected to Q-PCR. The relative enrichment of ChIP DNA was calculated relative to the input DNA. Each experiment was performed at least three times independently.

**RNA Immunoprecipitation**

The EZ-Magna RIP™ RNA-Binding Protein Immunoprecipitation Kit (17-701, Sigma-Aldrich) was used according to the manufacturer’s instructions. AGO immunoprecipitation was performed to explore whether miRNA regulates gene expression through the formation of the RNA-induced silencing complex (RISC). LX-2 cells were lysed in RIP Lysis Buffer [100 mM KCl, 5 mM MgCl2, 10 mM HEPES-NaOH, 0.5% Triton X-100,1 mM dithiothreitol (DTT), 0.2 U/ul RNase OUT (TaKaRa, 2313A), EDTA-free Protease Inhibitor Cocktail (APEXBIO, K1009-1), pH 7.4)] for 30 min on ice and the lysates were cleared by centrifugation (16,000 g) for 10 min at 4 °C. One hundred microliters of the supernatant were used as input, and the rest of the supernatant was incubated with anti-AGO2 antibody (2897, Cell Signaling Technology) or normal IgG coupled Magnetic Beads Protein A/G under constant rotation overnight at 4°C. The beads were recovered by gentle centrifugation and washed five times in RIP Wash Buffer [(50 mM Tris-HCl pH 7.4,250 mM NaCl, 1 mM MgCl2, 0.05% NP-40, 20 mM EDTA, 1 mM DTT, 0.2 U/ul RNase OUT (TaKaRa, 2313A)].Thirty percent of the immunoprecipitate was set aside for Western blot analysis, and the rest was used for RNA purification and detection by qRT-PCR. RNA was extracted using TRIzol reagent (15596018, Ambion) after digestion treatment with protease K Solution (I4333793, Invitrogen™).

**Statistical analysis**

Data are shown as the mean ± standard error of the mean (SEM) and are representative of at least three independent experiments. The two-tailed Student t test was used to compare two groups and identify significant differences mean values, and one-way ANOVA was used to compare more than two groups. GraphPad Prism 7 (GraphPad, San Diego, CA) was used to perform statistical analyses. P values of ≤0.05 were considered statistically significant.

**SUPPLEMENTARY TABLE 1**

Sequences used in this article.:

| **Items** | **Sequences (5’-3’)** |
| --- | --- |
| Human H19 Forward  Human H19 Reverse | ACTCAGGAATCGGCTCTGGAA  CTGCTGTTCCGATGGTGTCTT |
| Mouse H19 Forward  Mouse H19 Reverse | CAGAGCAAAGGCATCGCAAA  CTCCCCTTTATCCGACCAGC |
| Human JNK1 Forward  Human JNK1 Reverse | TGTGTGGAATCAAGCACCTTC  AGGCGTCATCATAAAACTCGTTC |
| GAPDH Forward  GAPDH Reverse | ACCACAGTCCATGCCATCAC  TCCACCACCCTGTTGCTGTA |
| miR-19a-3p Forward  miR-19a-3p Reverse | GCGGGCGGGTGTGCAAATCTAT  TCAACTGGTGTCGTGGAGTCGGC |
| miR-19b-3p Forward  miR-19b-3p Reverse | GCGGGCTGTGCAAATCCATGCAA  TCAACTGGTGTCGTGGAGTCGGC |
| miR-130a-3p Forward  miR-130a-3p Reverse | AGGAGCCAGTGCAATGTTAAAAGG  TCAACTGGTGTCGTGGAGTCGGC |
| miR-130b-3p Forward  miR-130b-3p Reverse | ACCGCCCAGTGCAATGATGAA  TCAACTGGTGTCGTGGAGTCGGC |
| miR-301a-3p Forward  miR-301a-3p Reverse | GCAAGCGGGCAGTGCAATAGTAT  TCAACTGGTGTCGTGGAGTCGGC |
| miR-491-5p Forward  miR-491-5p Reverse | GCAAGCATTAGTGGGGAACC  TCAACTGGTGTCGTGGAGTCGGC |
| miR-148a-3p Forward  miR-148a-3p Reverse | GAACGGGCGAGTCAGTGC  TCAACTGGTGTCGTGGAGTCGGC |
| miR-148b-3p Forward  miR-148b-3p Reverse | GCAAGCGGGTCAGTGCATCA  TCAACTGGTGTCGTGGAGTCGGC |
| U6 Forward  U6 Reverse | CTAAAATTGGAACGATACAG  AAATATGGAACGCTTCAC |
| Human c-Jun siRNA | AGTCATGAACCACGTTAAC |
| H19 pulldown probe | CTGCTGTTCCGATGGTGTCTTTGATGTTGGGCTGATGAGGTCTGGTTCCT |
| Luc-JNK1-3’UTR | CGTAAGGAAAACAGAAGTCCTAATTTCAAACTGACTGCTCTTCGTTAAGTGCTCTTAAGGAGAGTCTAGTAACAGTAACACTTTCTGGCCATTTCTAGTTTAGATTCTCTTCGTTACTGAAACTTTTGAGAAATATTACCTGTGGATTAATTTTGCACAATGTTCTATTCTCATAATGACTTACAAATTAAACTAGGTTTTTATTGAACTACCTCACACTAATTTTCTATGCTTTCCCAAGTAAGCTGTTGCCCTGTTAGATCTTTACTGAGTGAATTATAAATGTGTGTTAAATACTTTCTAGCCAATGTTGACACAATACCAGTAAGTATGTAAAGTATATACCTTAC |
| Luc-JNK1-3’UTR-mut | CGTAAGGAAAACAGAAGTCCTAATTTCAAACTGACTGCTCTTCGTTAAGTGCTCTTAAGGAGAGTCTAGTAACAGTAACACTTTCTGGCCATTTCTAGTTTAGATTCTCTTCGTTACTGAAACTTTTGAGAAATATTACCTGTGGATTAATTAACGTGAATGTTCTATTCTCATAATGACTTACAAATTAAACTAGGTTTTTATTGAACTACCTCACACTAATTTTCTATGCTTTCCCAAGTAAGCTGTTGCCCTGTTAGATCTTTACTGAGTGAATTATAAATGTGTGTTAAATACTTTCTAGCCAATGTTGACACAATACCAGTAAGTATGTAAAGTATATACCTTAC |
| H19-sponge site 1  (luciferase) | CACCACATCATCCCAGAGCTGAGCTCCTCCAGCGGGATGACGCCGTCCCCACCACCTCCCTCTTCTTCTTTTTCATCCTTCTGTCTCTTTGTTTCTGAGCTTTCCTGTCTTTCCTTTTTTCTGAGAGATTCAAAGCCTCCACGACTCTGTTTCCCCCGTCCCTTCTGAATTTAATTTGCACTAAGTCATTTGCACTGGTTGGAGTTGTGGAGACGGCCTTGAGTCTCAGTACGAGTGTGCGTGAGTGTGAGCCACCTTGGCAAGTGCCTGTGCAGGGCCCGGCCGCCCTCCATCTGGGCCGGGTGACTGGGCGCCGGCTGTGTGCCCGAGGCCTCACCCTGCCCTCGCCT |
| H19-sponge site 1-mut  (luciferase) | CACCACATCATCCCAGAGCTGAGCTCCTCCAGCGGGATGACGCCGTCCCCACCACCTCCCTCTTCTTCTTTTTCATCCTTCTGTCTCTTTGTTTCTGAGCTTTCCTGTCTTTCCTTTTTTCTGAGAGATTCAAAGCCTCCACGACTCTGTTTCCCCCGTCCCTTCTGAATTTAAAAACGTGATTCAGTAAACGTGTGGTTGGAGTTGTGGAGACGGCCTTGAGTCTCAGTACGAGTGTGCGTGAGTGTGAGCCACCTTGGCAAGTGCCTGTGCAGGGCCCGGCCGCCCTCCATCTGGGCCGGGTGACTGGGCGCCGGCTGTGTGCCCGAGGCCTCACCCTGCCCTCGCCT |
| H19-sponge site 2  (luciferase) | CACCACATCATCCCAGAGCTGAGCTCCTCCAGCGGGATGACGCCGTCCCCACCACCTCCCTCTTCTTCTTTTTCATCCTTCTGTCTCTTTGTTTCTGAGCTTTCCTGTCTTTCCTTTTTTCTGAGAGATTCAAAGCCTCCACGACTCTGTTTCCCCCGTCCCTTCTGAATTTAATTTGCACTAAGTCATTTGCACTGGTTGGAGTTGTGGAGACGGCCTTGAGTCTCAGTACGAGTGTGCGTGAGTGTGAGCCACCTTGGCAAGTGCCTGTGCAGGGCCCGGCCGCCCTCCATCTGGGCCGGGTGACTGGGCGCCGGCTGTGTGCCCGAGGCCTCACCCTGCCCTCGCCT |
| H19-sponge site 2-mut  (luciferase) | CACCACATCATCCCAGAGCTGAGCTCCTCCAGCGGGATGACGCCGTCCCCACCACCTCCCTCTTCTTCTTTTTCATCCTTCTGTCTCTTTGTTTCTGAGCTTTCCTGTCTTTCCTTTTTTCTGAGAGATTCAAAGCCTCCACGACTCTGTTTCCCCCGTCCCAAGACTTAAATTAAACGTGTAAGTCATTTGCACTGGTTGGAGTTGTGGAGACGGCCTTGAGTCTCAGTACGAGTGTGCGTGAGTGTGAGCCACCTTGGCAAGTGCCTGTGCAGGGCCCGGCCGCCCTCCATCTGGGCCGGGTGACTGGGCGCCGGCTGTGTGCCCGAGGCCTCACCCTGCCCTCGCCT |
| CHIP-JUN-sense  CHIP-JUN-antisense | CAGTGAGCCCATCTCCCAG  CTGGGAGATGGGCTCACTG |
| Collagen 1 Forward  Collagen 1 Reverse | GAGGGCCAAGACGAAGACATC  CAGATCACGTCATCGCACAAC |
| α-SMA Forward  α-SMA Reverse | CGTGGCTATTCCTTCGTTAC  TGCCAGCAGACTCCATCC |

**SUPPLEMENTARY DISCUSSION**

Liver fibrosis is a common process of chronic liver diseases such as viral hepatitis, alcoholic liver disease and fatty liver. Activation of HSCs is the central event during hepatic fibrosis [2], but the mechanisms underlying the perpetual activation of HSCs remain elusive. For example, the activation process of HSCs is divided into two stages: initial activation and permanent activation. HSCs in the initial activated state can be cleared by physiological processes such as apoptosis, aging and restoration of inactivation, indicating that the activation of HSCs is dynamically reversible [2]. It still remains to be fully understood how HSCs maintain the permanent activation state, which may be the key to reversing liver fibrosis.

H19, a well-known imprinted long noncoding RNA (lncRNA), has been verified to be increased in cholestatic livers [3-6]. Few studies have focused on HSCs to explore the role of H19 during hepatic fibrosis while many studies have focused on hepatocytes and cholangiocytes [3-6]. In this study, we determined the upregulation of H19 in activated HSCs and confirmed that H19 was significantly increased in acute liver injury (constructed by bile duct ligation) and chronic liver injury (induced by intraperitoneal injection of CCL4) by quantitative RT-PCR. In turn, H19 overexpression in vitro further exaggerated HSC activation, while downregulation of H19 reduced the protein levels of α-SMA and Collagen 1 in activated LX-2 cells. Moreover, Li et al. demonstrated that downregulation of H19 through adenovirus of H19 shRNA markedly reduced hepatobiliary injury and fibrosis [4]. Song et al. have even directly elucidated the role of H19 in BDL-induced liver fibrosis by using H19−/− mice, and H19 deficiency dramatically prevented cholestatic liver fibrosis [3]. These results indicated a positive relationship between H19 and liver fibrosis both in vitro and in vivo.

Regarding the source of H19 in activated HSCs, a previous study showed that both quiescent and activated HSCs absorb cholangiocyte-derived H19 [6]. Transcriptional regulation controlled by hypoxia inducible factor-1α (HIF-1α) also promoted the production of H19 in activated HSCs [7]. Here we found that c-Jun directly drove the transcription of H19 by targeting the promoter region in activated HSCs. We predicted the binding sites of c-Jun in the promoter region of the H19 gene by biogenic analysis and confirmed the binding between them by a luciferase assay (Figure 2C). Additionally, a chromatin immunoprecipitation (ChIP) assay was performed by using anti-c-Jun or anti-IgG antibody to further verify the direct binding between total c-Jun protein and the H19 promoter region. Quantitative RT‒PCR analysis revealed that the promoter fragment of the H19 gene was much more captured by c-Jun immunoprecipitates than by control IgG immunoprecipitates, and the increase in c-Jun coprecipitated H19 promoter fragments was significantly enlarged with the overexpression of c-Jun protein (OE-c-Jun), indicating direct binding and a positive relationship between total c-Jun and the H19 promoter region (Figure 2D). Indeed, it has been recognized that the transcription factor c-Jun requires N-terminal phosphorylation or C-terminal dephosphorylation to turn on transactivation activity and increase DNA binding affinities, respectively [8, 9]. Combined with the fact that c-Jun is one of the downstream substrates of JNK and is phosphorylated by JNK1 at the serines 63 and 73 of N-termini [8, 10], which results in a robust induction of c-Jun transactivation activity, we could conclude that c-Jun phosphorylated by JNK1 actually binds to the H19 promoter and promotes H19 expression in active HSCs during liver fibrosis. As phosphorylated c-Jun is difficult to directly overexpress in cells, we did not further attempt to increase c-Jun phosphorylation in cells to check its binding with the H19 promoter. However, the c-Jun antibody we used in the ChIP assay recognized both phosphorylated and nonphosphorylated c-Jun. Therefore, although we have not been able to verify its binding to the H19 promoter region by overexpression of phospho-c-Jun, the literature review and the upregulation of JNK1 and phosphorylated JNK1 in liver fibrosis give us good reason to conclude that it is phospho-c-Jun binding to the H19 promoter. It remains unclear how much c-Jun, HIF-1α or cholangiocyte-derived H19 contributes to the increase in H19 in HSCs, which needs to be further studied.

When exploring the regulatory network of H19 in HSC activation, we observed that H19 was mainly located in the cytoplasm of HSCs, indicating that H19 is more likely to function through a ceRNA mechanism, for example, acting as a molecular sponge to hijack microRNAs. We further determined that miR-19a-3p and miR-19b-3p were the microRNAs mainly sponged by H19 in LX-2 cells. Nevertheless, the expression levels of miR-19a/b-3p in LX-2 cells were not affected by the H19 sponge, whereas miR-19a/b-3p levels were downregulated in the fibrotic livers. Differential gene analysis based on the GEO databases also showed that miR-19a/b-3p was generally downregulated in liver diseases of various causes, such as HBV, HCV, alcoholic liver disease and NASH. This finding implied that although the H19 sponge did not change miR-19a/b-3p levels in LX-2 cells, it might affect the function of miR-19a/b-3p to some extent. In addition, miR-19a/b-3p might be decreased in other cell types such as hepatocytes and Kupffer cells, during liver fibrosis. Another possibility is that regulators other than H19 sponges must affect the transcription, processing, maturation or secretion of miR-19a/b-3p during liver fibrosis. These clues deserve more attention in future studies. Importantly, this finding confirmed that miR-19a/b-3p participates in the process of hepatic fibrosis, which has rarely been investigated before.

To explore the potential role of miR-19a/b-3p during liver fibrosis, we overexpressed miR-19a-3p and miR-19b-3p in the fibrotic liver through tail vein injection of miR-19a/b-3p-overexpressing lentivirus. We found that both miR-19a-3p and miR-19b-3p have a surprisingly remarkable effect on restoring the pathological phenotypes of liver fibrosis in both BDL and CCL4 mice. This result indicated that miR-19a/b-3p plays an important antifibrotic role in fibrotic livers. Previous studies have shown that miRNAs are important factors that interfere with hepatic fibrogenesis [11]. For instance, miR-155 [12, 13], miR-199a [14] and miR-34a [15] inhibit key signaling pathways that promote HSC activation and liver fibrosis, while let-7 [16], miR-29b [17] and miR-133a [18] have distinct antifibrotic effects. miR-19b also displayed an inhibitory effect in HSC-mediated fibrogenesis, but the underlying mechanism has not yet been determined [19]. Herein, we provided an interesting and important finding that JNK1 was among the potential downstream target genes of miR-19a/b-3p. Further experiments verified that miR-19a-3p and miR-19b-3p directly targeted the 3’UTR of JNK1 and suppressed the expression of JNK1 in LX-2 cells. This negative interaction between miR-19a/b-3p and JNK1 was also demonstrated in fibrotic livers. JNK signaling is crucial for cell death, survival, differentiation, proliferation and tumorigenesis in the liver [20]. In nonparenchymal liver cells, such as hepatic macrophages (Kupffer cells) and HSCs, JNK is involved in inflammation and fibrosis [21, 22]. The distinct functions of the JNK1 and JNK2 isoforms in the pathogenesis of liver diseases have been characterized in two independent murine models (JNK1-/- and JNK2-/- mice), of which JNK1-/- but not JNK2-/- mice were well protected from liver fibrosis. In addition, JNK1 in HSCs but not in hepatocytes significantly contributes to liver fibrosis development, and was identified as an essential profibrogenic kinase in HSC activation [23].

Therefore, the regulatory axis of miR-19a/b-3p/JNK1 we found might play an important role in the activation of HSCs during liver fibrosis. Combining the results above, we identified a feedback loop consisting of c-Jun, H19, miR-19a/b-3p and JNK1 during HSC activation and hepatic fibrosis. This feedback loop of c-Jun/H19/miR-19/JNK1/c-Jun further amplified the cascade of HSC activation and probably promoted the transition or change of HSCs from the initial activation state to the permanent activation state. Our findings reinforce the remarkable complexity and plasticity of HSC activation, and underscore the value of epigenetic regulation in promoting the development of novel therapies for liver fibrosis.

**SUPPLEMENTARY REFERENCE**

1. Zhao, J., et al., *The HIF-1A/miR-17-5p/PDCD4 axis contributes to the tumor growth and metastasis of gastric cancer.* Signal Transduct Target Ther, 2020. **5**(1): p. 46.

2. Tsuchida, T. and S.L. Friedman, *Mechanisms of hepatic stellate cell activation.* Nat Rev Gastroenterol Hepatol, 2017. **14**(7): p. 397-411.

3. Song, Y., et al., *H19 promotes cholestatic liver fibrosis by preventing ZEB1-mediated inhibition of epithelial cell adhesion molecule.* Hepatology, 2017. **66**(4): p. 1183-1196.

4. Li, X., et al., *The role of long noncoding RNA H19 in gender disparity of cholestatic liver injury in multidrug resistance 2 gene knockout mice.* Hepatology, 2017. **66**(3): p. 869-884.

5. Li, X., et al., *Cholangiocyte-derived exosomal long noncoding RNA H19 promotes cholestatic liver injury in mouse and humans.* Hepatology, 2018. **68**(2): p. 599-615.

6. Liu, R., et al., *Cholangiocyte-Derived Exosomal Long Noncoding RNA H19 Promotes Hepatic Stellate Cell Activation and Cholestatic Liver Fibrosis.* Hepatology, 2019. **70**(4): p. 1317-1335.

7. Xia, S., et al., *Dihydroartemisinin regulates lipid droplet metabolism in hepatic stellate cells by inhibiting lncRNA-H19-induced AMPK signal.* Biochem Pharmacol, 2021. **192**: p. 114730.

8. Smeal, T., et al., *Oncogenic and transcriptional cooperation with Ha-Ras requires phosphorylation of c-Jun on serines 63 and 73.* Nature, 1991. **354**(6353): p. 494-496.

9. Boyle, W.J., et al., *Activation of protein kinase C decreases phosphorylation of c-Jun at sites that negatively regulate its DNA-binding activity.* Cell, 1991. **64**(3): p. 573-584.

10. Hibi, M., et al., *Identification of an oncoprotein- and UV-responsive protein kinase that binds and potentiates the c-Jun activation domain.* Genes Dev, 1993. **7**(11): p. 2135-2148.

11. Calvopina, D.A., et al., *MicroRNA Sequencing Identifies a Serum MicroRNA Panel, Which Combined With Aspartate Aminotransferase to Platelet Ratio Index Can Detect and Monitor Liver Disease in Pediatric Cystic Fibrosis.* Hepatology, 2018. **68**(6): p. 2301-2316.

12. Blaya, D., et al., *Expression of microRNA-155 in inflammatory cells modulates liver injury.* Hepatology, 2018. **68**(2): p. 691-706.

13. Bala, S., et al., *The pro-inflammatory effects of miR-155 promote liver fibrosis and alcohol-induced steatohepatitis.* J Hepatol, 2016. **64**(6): p. 1378-1387.

14. Lee, C.G., et al., *Farnesoid X receptor protects hepatocytes from injury by repressing miR-199a-3p, which increases levels of LKB1.* Gastroenterology, 2012. **142**(5): p. 1206-1217.e7.

15. Song, L., et al., *Pterostilbene prevents hepatocyte epithelial-mesenchymal transition in fructose-induced liver fibrosis through suppressing miR-34a/Sirt1/p53 and TGF-β1/Smads signalling.* Br J Pharmacol, 2019. **176**(11): p. 1619-1634.

16. Matsuura, K., et al., *Circulating let-7 levels in plasma and extracellular vesicles correlate with hepatic fibrosis progression in chronic hepatitis C.* Hepatology, 2016. **64**(3): p. 732-745.

17. Roderburg, C., et al., *Micro-RNA profiling reveals a role for miR-29 in human and murine liver fibrosis.* Hepatology, 2011. **53**(1): p. 209-218.

18. Roderburg, C., et al., *miR-133a mediates TGF-β-dependent derepression of collagen synthesis in hepatic stellate cells during liver fibrosis.* J Hepatol, 2013. **58**(4): p. 736-42.

19. Lakner, A.M., et al., *Inhibitory effects of microRNA 19b in hepatic stellate cell-mediated fibrogenesis.* Hepatology, 2012. **56**(1): p. 300-310.

20. Seki, E., D.A. Brenner, and M. Karin, *A liver full of JNK: signaling in regulation of cell function and disease pathogenesis, and clinical approaches.* Gastroenterology, 2012. **143**(2): p. 307-320.

21. Das, M., et al., *Induction of hepatitis by JNK-mediated expression of TNF-alpha.* Cell, 2009. **136**(2): p. 249-260.

22. Solinas, G., et al., *JNK1 in hematopoietically derived cells contributes to diet-induced inflammation and insulin resistance without affecting obesity.* Cell Metab, 2007. **6**(5): p. 386-397.

23. Zhao, G., et al., *Jnk1 in murine hepatic stellate cells is a crucial mediator of liver fibrogenesis.* Gut, 2014. **63**(7): p. 1159-1172.
