# Supplementary figures and images for: Identification of the c‐Jun/H19/miR‐19/JNK1 cascade during hepatic stellate cell activation
Source: Clin Transl Med. 2023 Mar 2;13(3):e1106. doi: 10.1002/ctm2.1106 (PMC9982076; doi:10.1002/ctm2.1106)

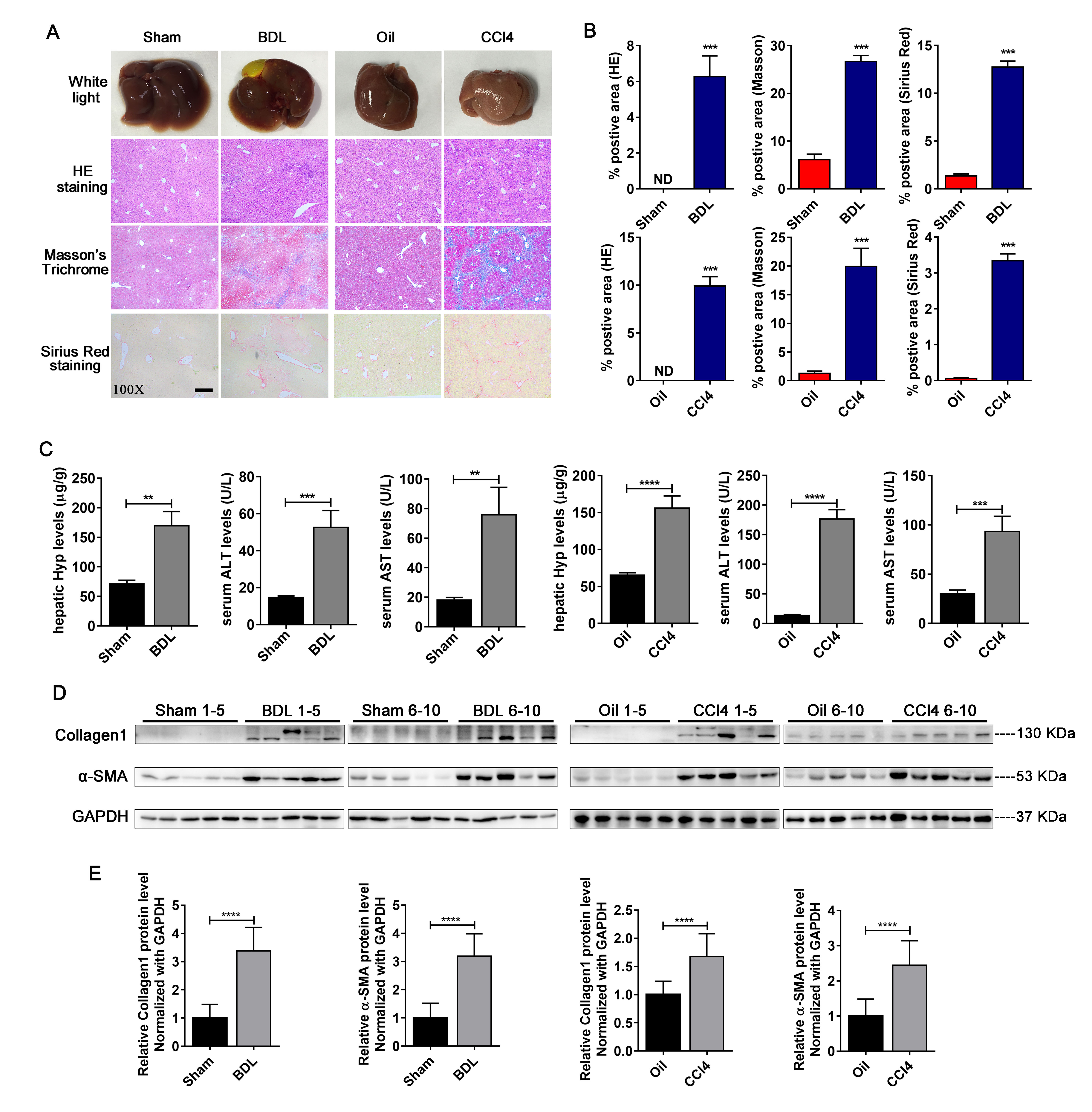

Supplement: Supplementary file 2 — Supporting Material [file CTM2-13-e1106-s008.jpg]

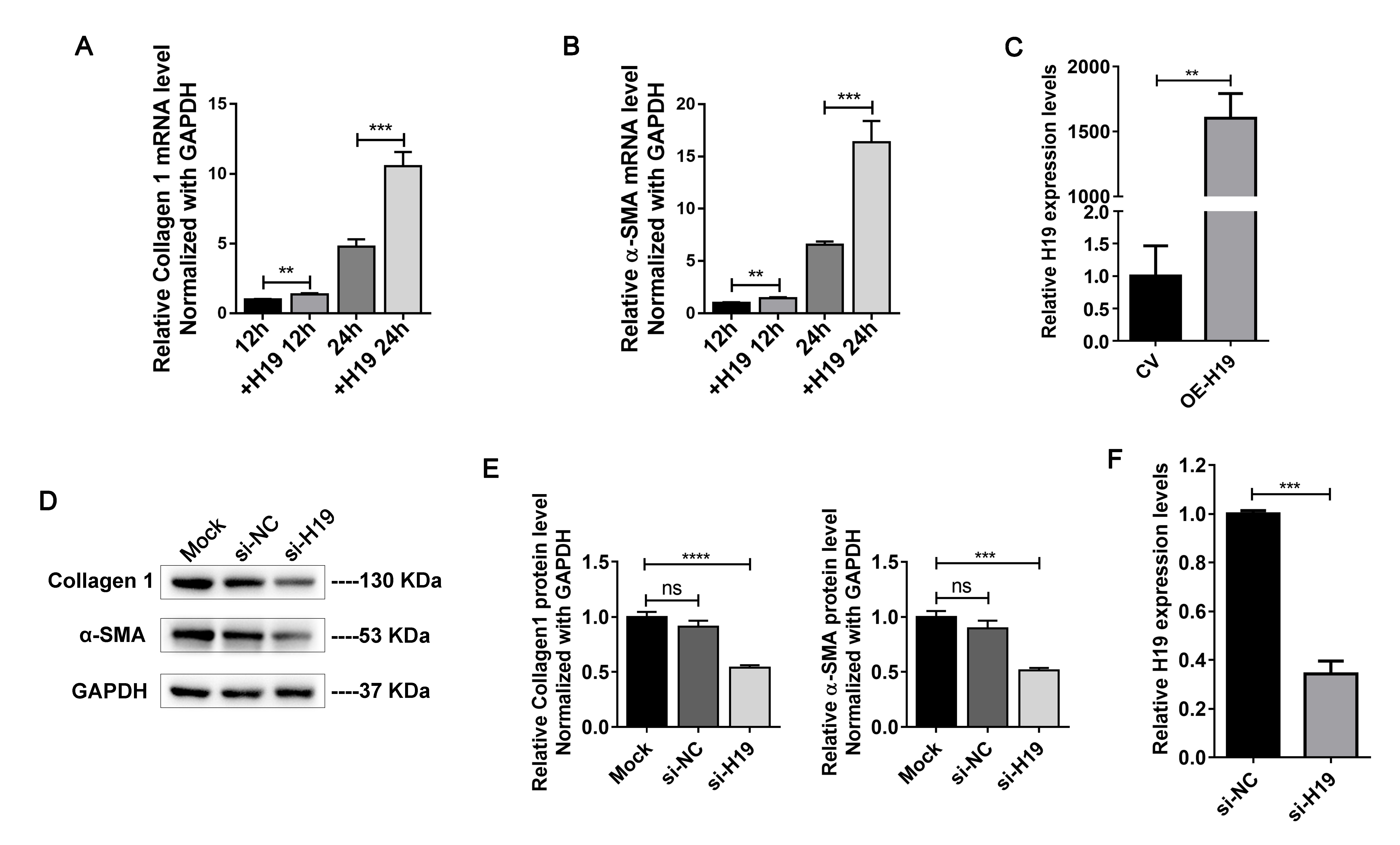

Supplement: Supplementary file 3 — Supporting Material [file CTM2-13-e1106-s002.jpg]

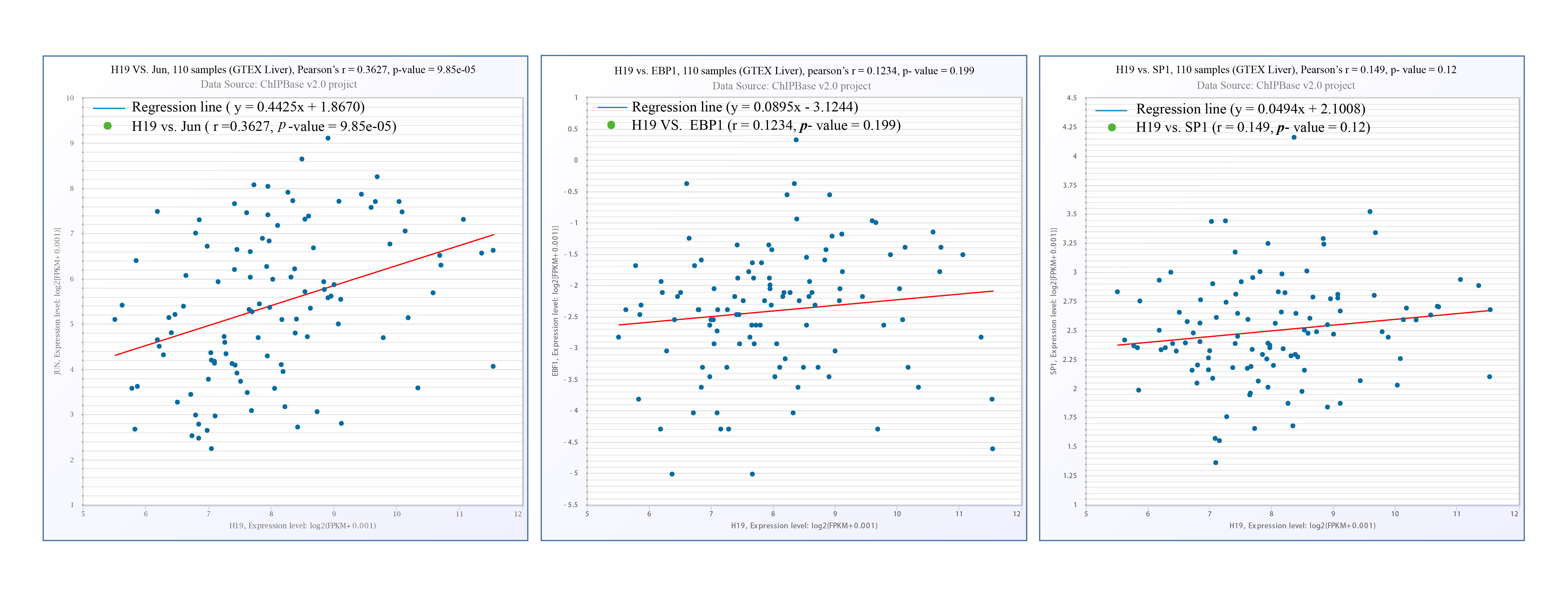

Supplement: Supplementary file 4 — Supporting Material [file CTM2-13-e1106-s004.jpg]

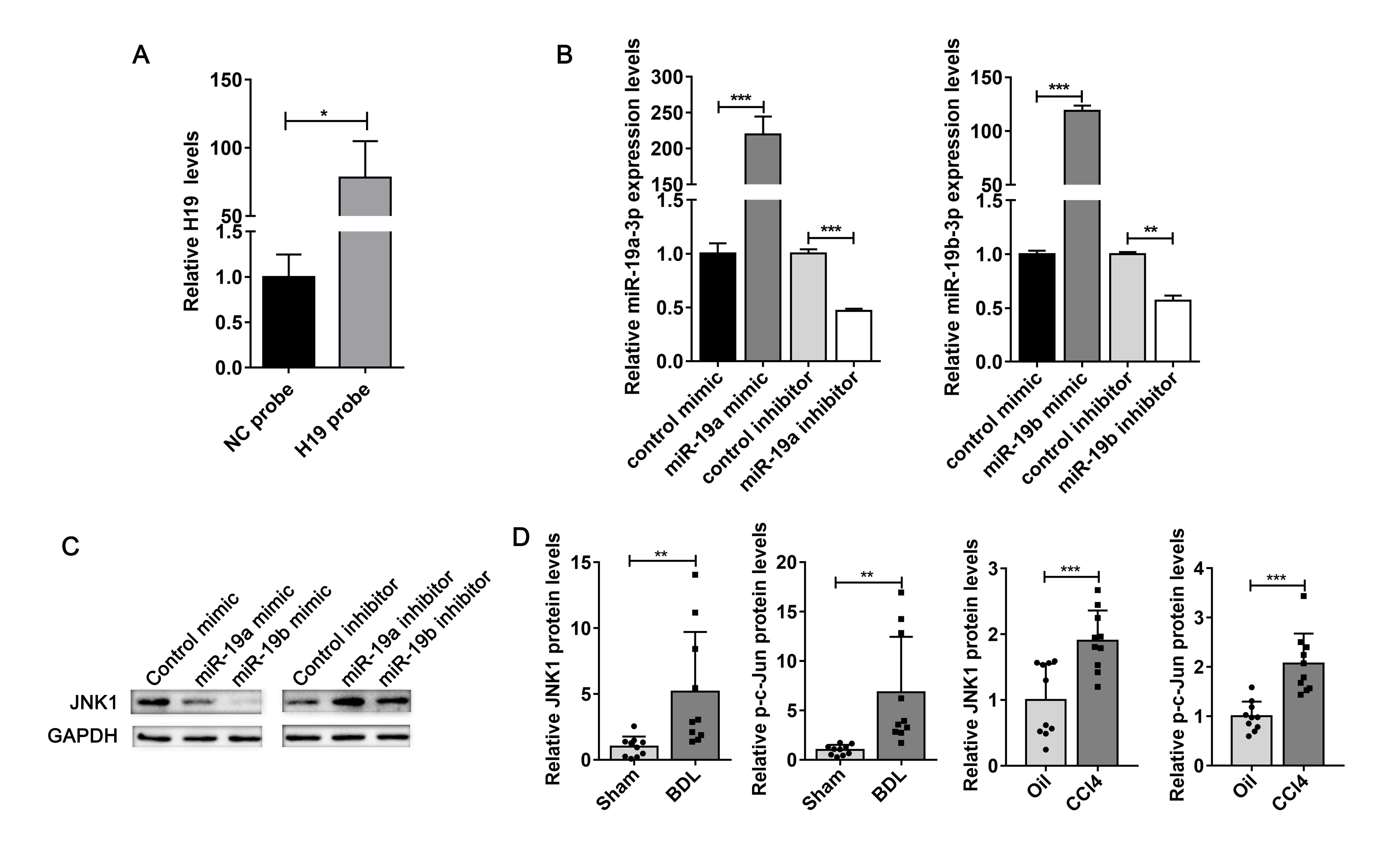

Supplement: Supplementary file 5 — Supporting Material [file CTM2-13-e1106-s007.jpg]

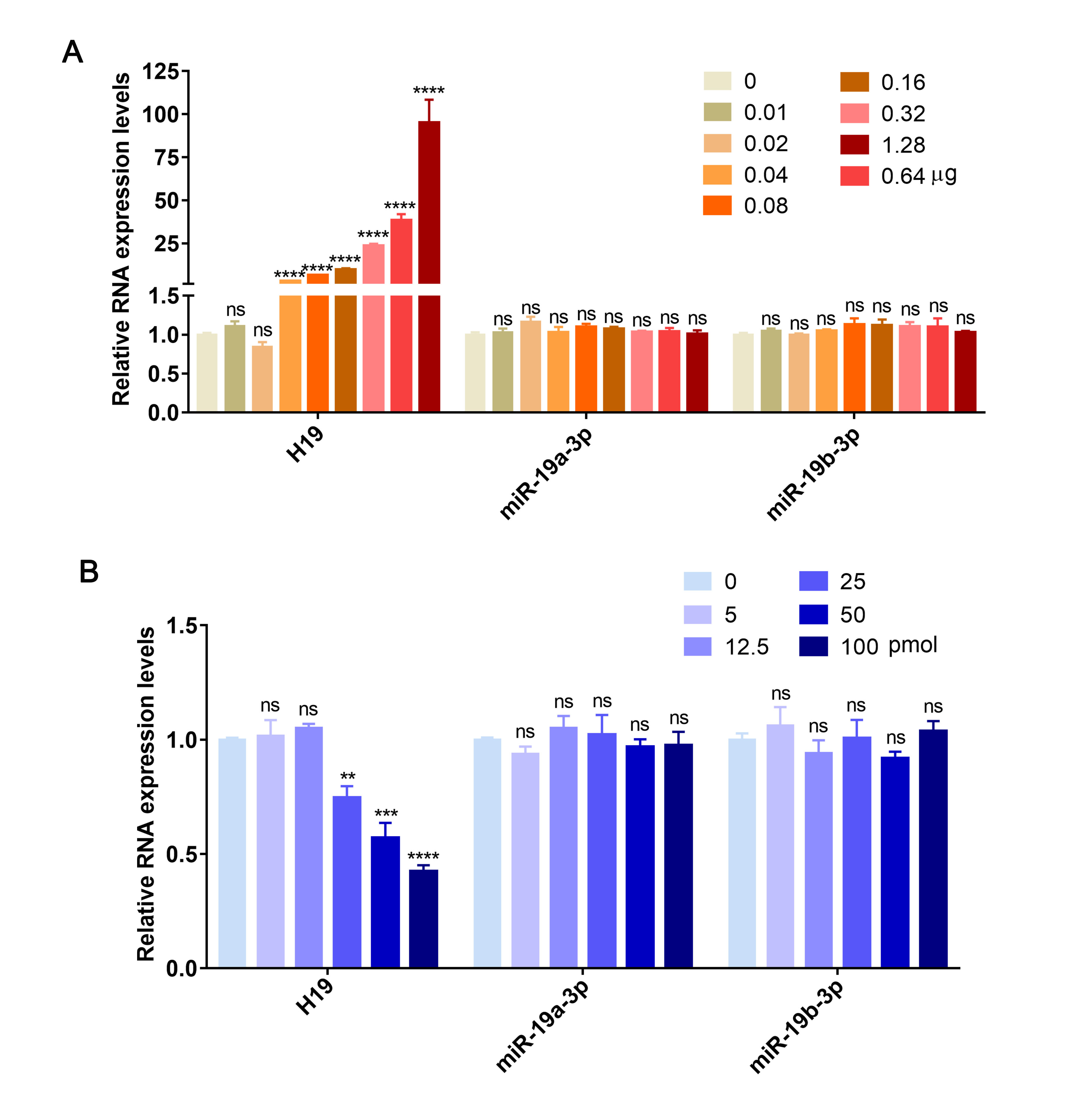

Supplement: Supplementary file 6 — Supporting Material [file CTM2-13-e1106-s003.jpg]

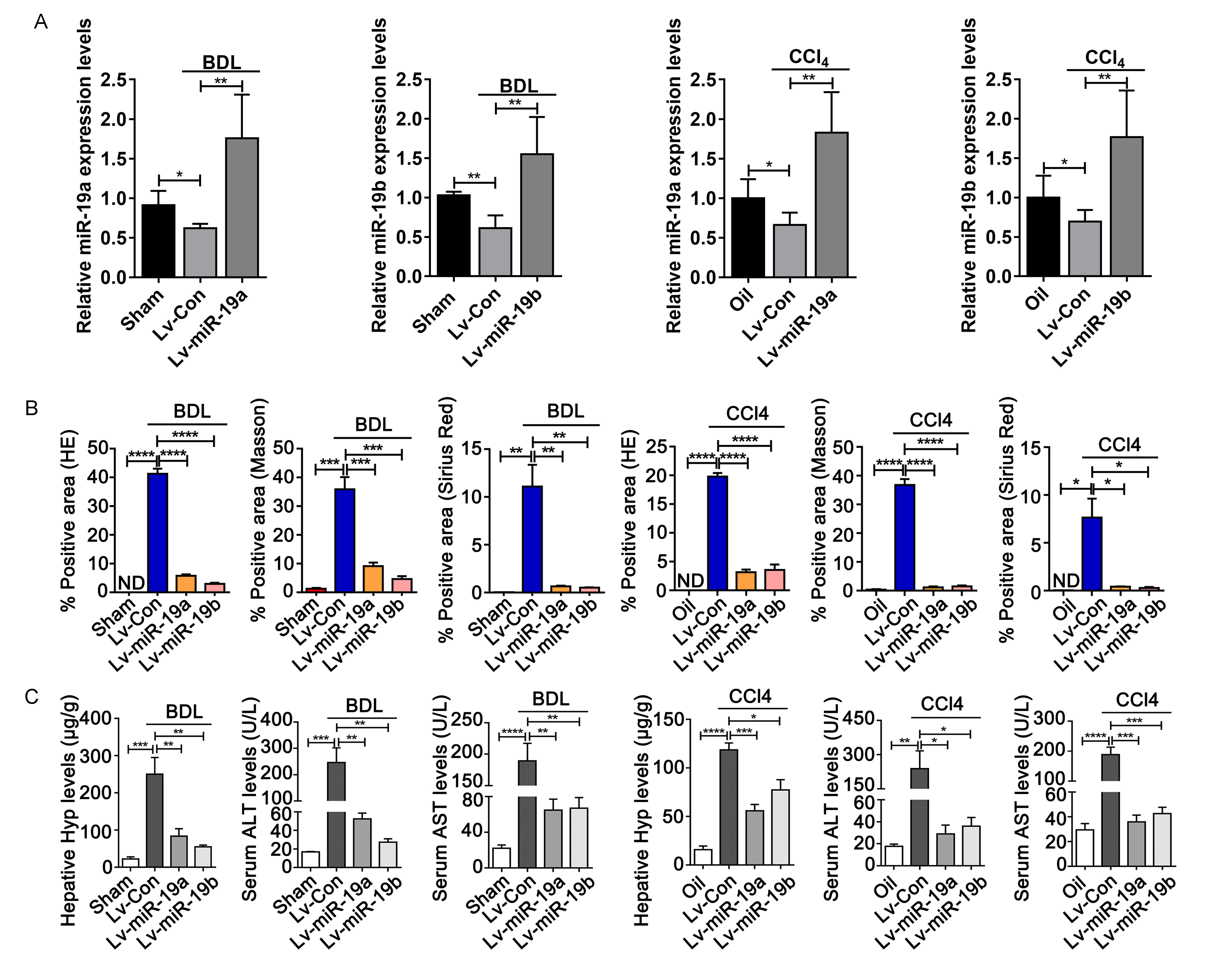

Supplement: Supplementary file 7 — Supporting Material [file CTM2-13-e1106-s001.jpg]

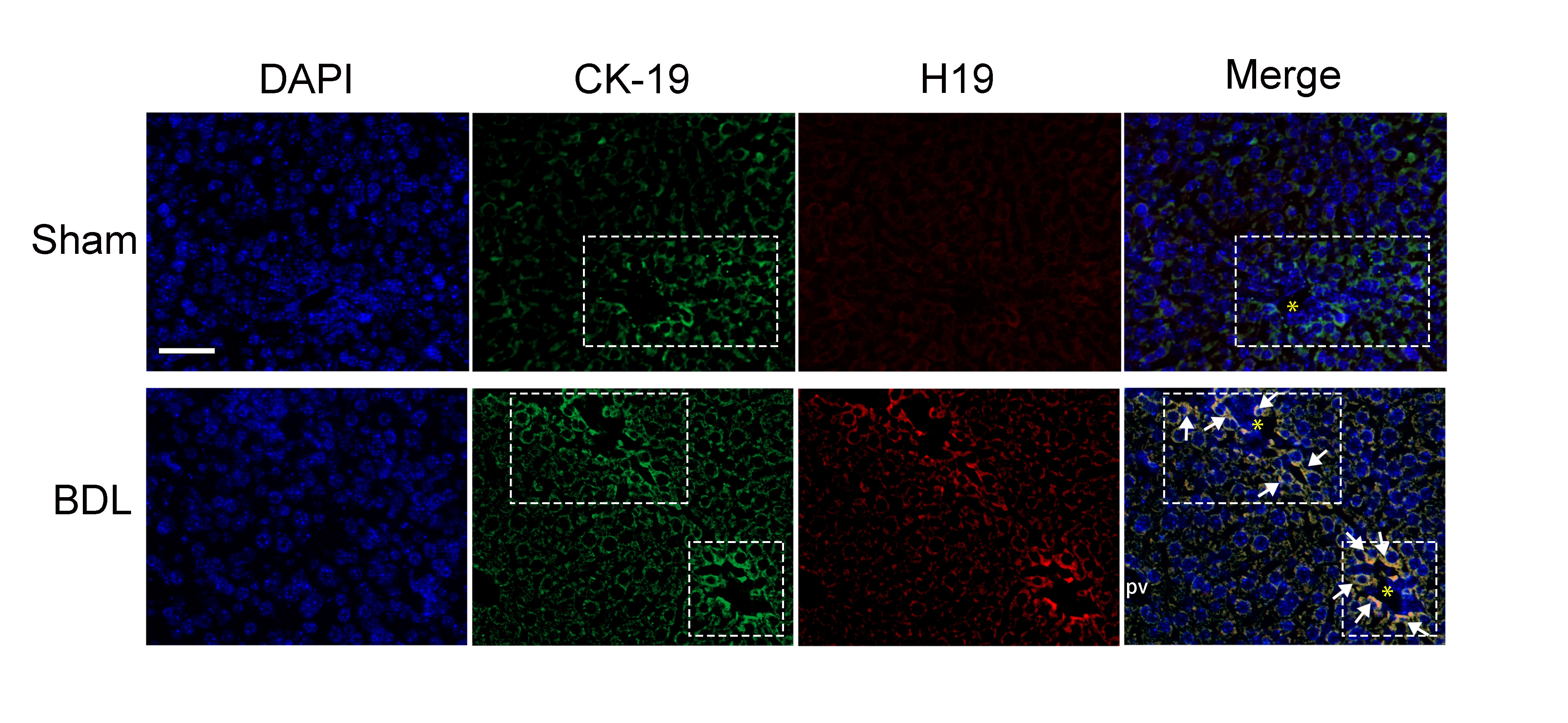

Supplement: Supplementary file 8 — Supporting Material [file CTM2-13-e1106-s005.jpg]
